# Prevalence of Non-Tuberculous Mycobacteria in Hospital Waters of Major Cities of Khuzestan Province, Iran

**DOI:** 10.3389/fcimb.2016.00042

**Published:** 2016-04-13

**Authors:** Azar Dokht Khosravi, Abdolrazagh Hashemi Shahraki, Mohammad Hashemzadeh, Rasa Sheini Mehrabzadeh, Ali Teimoori

**Affiliations:** ^1^Health Research Institute, Infectious and Tropical Diseases Research Center, Ahvaz Jundishapur University of Medical SciencesAhvaz, Iran; ^2^Department of Microbiology, School of Medicine, Ahvaz Jundishapur University of Medical SciencesAhvaz, Iran; ^3^Department of Epidemiology, Pasteur Institute of IranTehran, Iran; ^4^Student Research Committee, Ahvaz Jundishapur University of Medical SciencesAhvaz, Iran; ^5^Department of Virology, School of Medicine, Ahvaz Jundishapur University of Medical ScienceAhvaz, Iran

**Keywords:** *non-tuberculous mycobacteria*, polymerase chain reaction, restriction enzyme analysis, water samples

## Abstract

Non-tuberculous mycobacteria (NTM) are among the emerging pathogens in immunocompromised individuals including hospitalized patients. So, it is important to consider hospitals water supplies as a source for infection. The aim of this study was to determine the prevalence of NTM in the hospital aquatic systems of Khuzestan, South west of Iran. In total, 258 hospital water samples were collected and examined. After initial sample processing, sediment of each sample were inoculated into two Lowenstein-Jensen medium. The positive cultures were studied with phenotypic tests including growth rate, colony morphology, and pigmentation, with subsequent PCR- restriction enzyme analysis (PRA) and *rpoB* gene sequence analysis. Mycobacterial strains were isolated from 77 samples (29.8%), comprising 52 (70.1%) rapid growing, and 25 (32.4%) slow growing mycobacteria. Based on the overall results, *M. fortuitum* (44.1%) was the most common mycobacterial species in hospital water samples, followed by *M. gordonae* (*n* = 13, 16.8%) and *M. senegalense* (*n* = 5, 7.7%). In conclusion, current study demonstrated the NTM strains as one of the major parts of hospital water supplies with probable potential source for nosocomial infections. This finding also help to shed light on to the dynamics of the distribution and diversity of NTM in the water system of hospitals in the region of study.

## Introduction

Non-tuberculous mycobacteria (NTM) are widely present in the environment and are commonly isolated from environmental sources including water (Decker and Palmore, 2013). Several species of environmental mycobacteria have been known to be important human pathogens, and reports suggest that there is an increasing trend in the incidence of NTM disease (Moore et al., 2010; Donohue et al., 2015), and in particular, in immunocompromised patients (Parashar et al., 2004; Narang et al., 2009). In a recently published meta-analysis, high prevalence of NTM infections (10.2%) was reported among culture-positive cases of tuberculosis (TB) in Iran, which emphasizes on the role of NTM in human public health (Nasiri et al., 2015). Exposure to NTMs occurs primarily through human interactions with water (Donohue et al., 2015). In particular, NTM species can multiply in the numerous water sources, including waste water, surface water, ground water, and tap water (Edirisinghe et al., 2014). Mycobacteria are able to multiply within low nutrient environment particularly water pip systems and can survive in hospital hot water systems, and resist chlorination. Moreover, biofilm formation, amoeba-associated lifestyle, and resistance to chlorine have been recognized as important factors that contribute to the survival, colonization and persistence of NTM in water distribution systems (Vaerewijck et al., 2005; Castillo-Rodal et al., 2012; Falkinham, 2015; Iii, 2015). The incidence of NTM has been reported different from one setting to another, ranging from 40 (Shin et al., 2007) to 58% (Crago et al., 2014). Some aquatic systems such as hospital water lines are more suitable for colonization with mycobacteria and biofilm formation (Decker and Palmore, 2013). NTM are among the cause of opportunistic nosocomial infections in different clinical settings both in developed and developing countries, but there is little known about the isolation and identification of NTM in Iranian hospitals. So, the aim of this study was to determine the prevalence of NTM in the hospital aquatic systems of Khuzestan, Southwest of Iran, as the first survey on epidemiologic status and distribution of NTM species in hospital waters in the region of study.

## Materials and methods

### Sample collection

In total, 258 water samples were collected from different sites of teaching hospitals in the major cities of Khuzestan province (Ahvaz, Dezful and Abadan), from 2013 to 2015. These included tap water, dentistry unit water, haemodialysis center fluid, ventilator water, and drinking water tanks. Approximately one liter of each sample was collected in a sterile glass bottle, transferred to laboratory in an icebox and analyzed within 24 hrs. Chlorinated drinking water was the source of water tanks and dentistry unit water system, while sterile water used as water source of ventilator systems. Stranded procedure used to produce water for hemodialysis machines (CDC, 2003).

### Sample preparation and culture

The samples were processed and decontaminated as previously described (Peters et al., 1995). In brief, 500 ml of each water sample was filtered through a membrane filter (pore size 0.45 μm; Millipore, Bedford, U.S.A), and was decontaminated by 0.005% cetylpyridinium chloride for 30 min at room temperature. Two hundred μl of sediments were inoculated into two Lowenstein-Jensen (LJ) media (HiMedia, India), and incubated at 37 and 25°C for 2 months. The cultures were observed twice per week for growth rate, colony morphology, and pigmentation. Colonies were stained by the Ziehl-Neelsen method for the presence of acid fast bacilli (AFB). For acid fast positive colonies, standard phenotypic tests including niacin accumulation, nitrate reduction, catalase activity, iron uptake, and arylsulfatase activity were performed according to the standard instructional (Kent and Kubica, 1985), followed by molecular identification technique.

### Molecular identification

#### DNA extraction

Genomic DNA was extracted from colonies using lipase (Type VII; final concentration, 2 mg/ml [Sigma]), proteinase K (100 pg/ml) and 0.5% sodium dodecyl sulfate treatment as described by Neumann et al. (1997). The DNA was purified by phenol chloroform isoamyl alcohol and precipitated with isopropanol. The precipitate was washed in 70% ethanol, dehydrated and dissolved in 100 μl of Milli-Q water and stored in 20°C until use.

#### PCR restriction enzyme analysis (PRA)-based identification method

An approximately 441 bp fragment of the *hsp65* gene was amplified by PCR using two specific primers Tb11 (5′- ACCAACGATGGTGTGTCCAT-3′) and Tb12 (5′-CTTGTC GAACCGCA- TACCCT-3′). Genomic DNA of *M. fortuitum* ATCC 49404T and double distilled water were used as positive and negative controls, respectively. PCR products of *hsp65* gene were digested by the *Bst*EII and *Hae*III restriction enzyme according to previously described protocol (Telenti et al., 1993). The fragments were compared with those of patterns deposited in a free available database (http://app.chuv.ch/prasite, n.d.), for species identification.

#### Sequencing of rpoB gene

A 750-bp fragment of the *rpoB* gene was amplified using primers MycoF (5′- GGCAAGGTCACCCCGAAGGG-3′) and MycoR (5′-AGCGGCTGCTGGGTGATCATC- 3′) with instruction described by Adékambi et al. (2003). The cycling condition was 95°C for 1 min, followed by 30 cycles of 95°C for 30 s, 64°C for 30 s, and 72°C for 30 s and finalized with 72°C for 5 min.

#### Sequence data and phylogenetic analysis

The sequences of *rpoB* gene for each isolate were aligned separately and compared with all existing relevant sequences of mycobacteria recovered from GenBank database using JPhydit program (Jeon et al., 2005). Percentages of similarity between sequences of each gene were determined by comparing sequences to an in-house database of *rpoB* sequences. Phylogenetic trees were obtained from DNA sequences using the Neighbor-Joining (NJ) method and Kimura's two parameter (K2P) distance correction model with 1000 bootstrap replications supported by the MEGA 6 software (Tamura et al., 2007).

#### Nucleotide sequence accession numbers

The GenBank accession numbers of investigated isolates of NTM determined in this work are KU198411-KU198415, KU240541-KU240549, and KU212280 for *rpoB* gene.

## Statistical analysis

Data was shown through the simple predictive statistics, prevalence value and no any statistical method was used for comparing and prediction. Our proposed goal was completed with distribution and prevalence value of NTM in each location.

## Results

**Figure 1:**
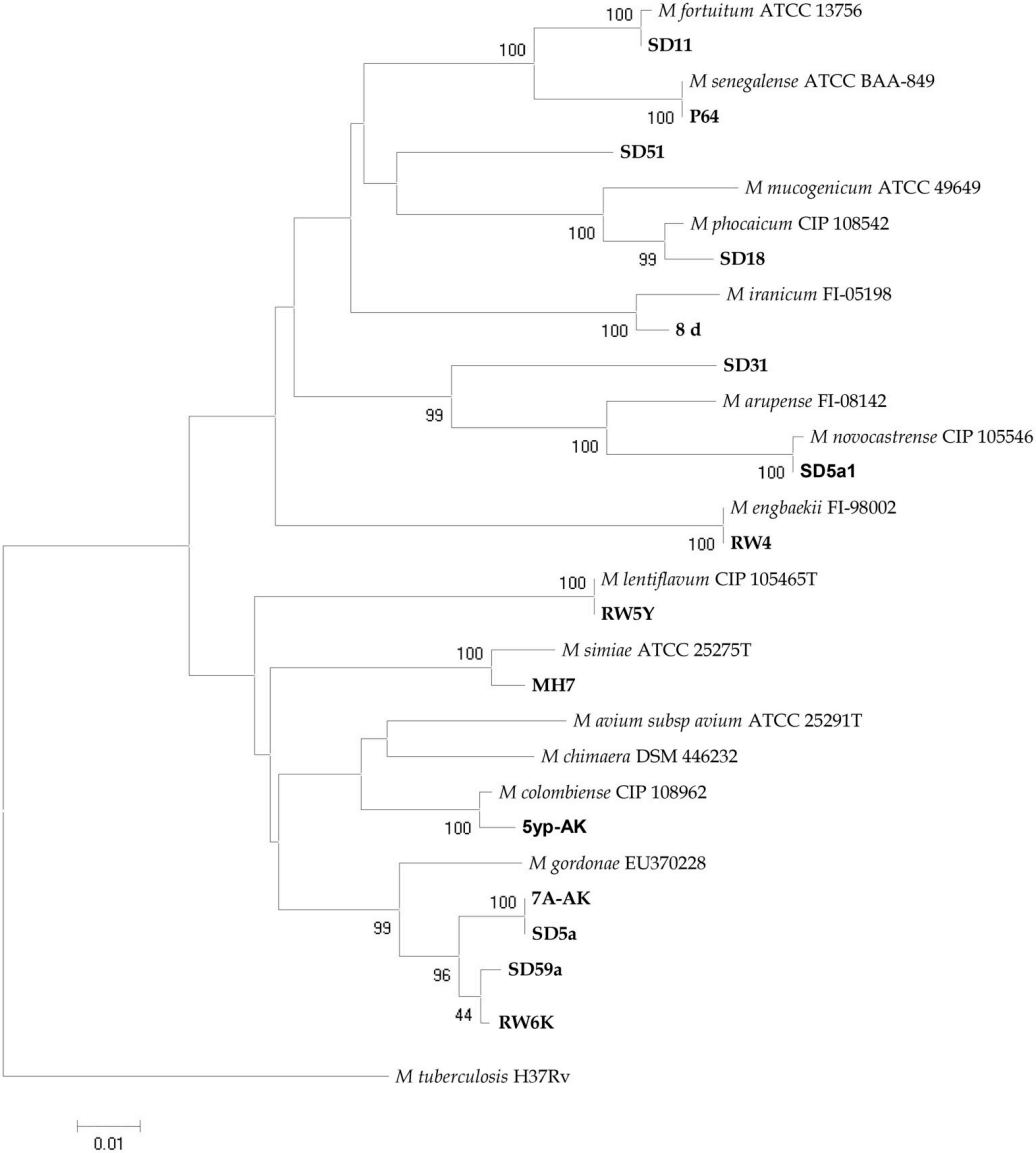
***rpoB* sequence-based phylogenetic tree of the isolates of NTM from water samples of hospitals with those of closely related species which computed by the NJ analyses and K2P model**. The support of each branch, as determined from 1000 bootstrap samples, is indicated by percentages at each node. Bar 0.01 substitutions per nucleotide position.

**Figure 2:**
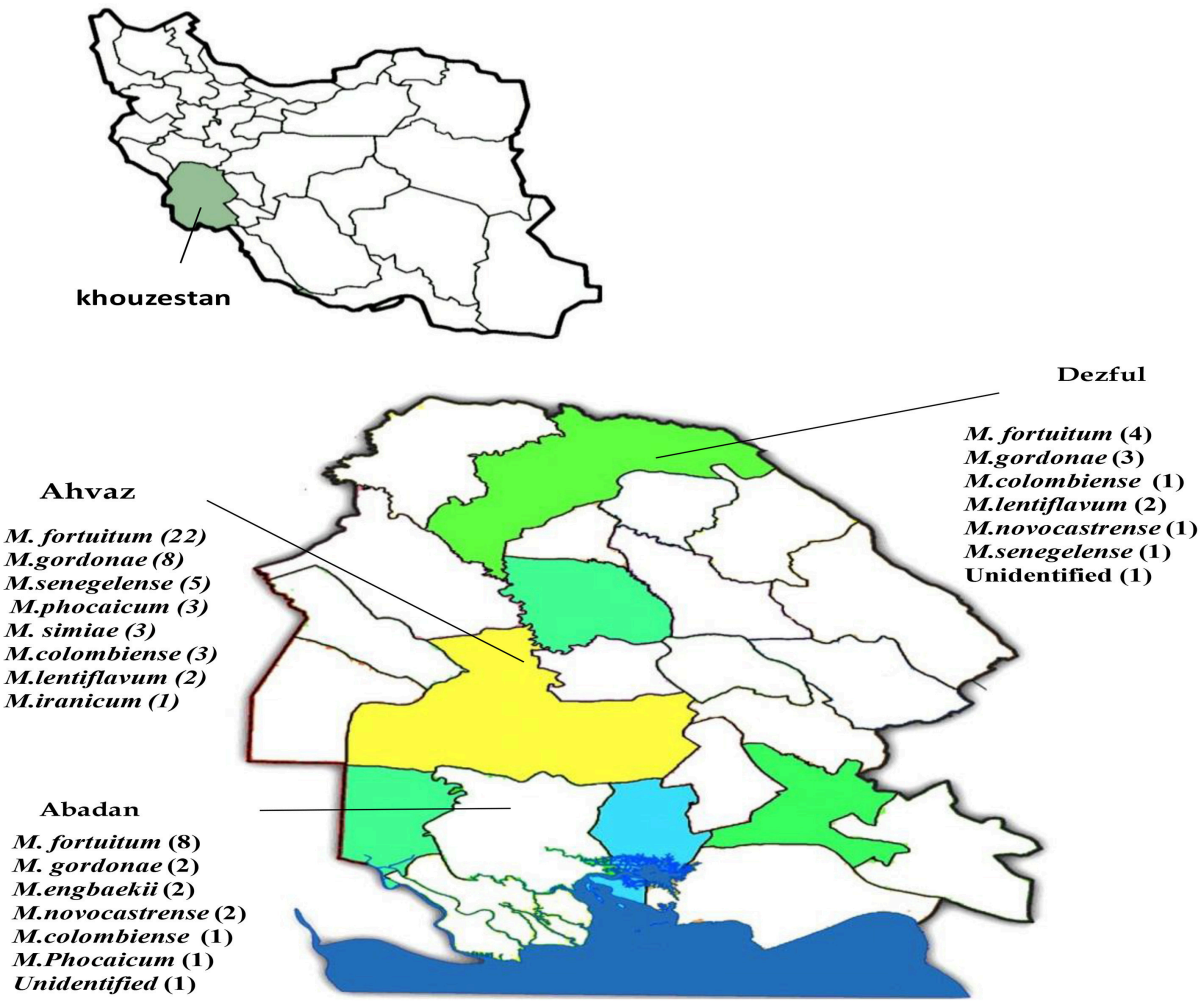
**Geographic distribution of NTM species among the hospitals of three area of Khuzestan**.

The analyzed water samples comprised: 180 from Ahvaz, 40 from Abadan, and 38 from Dezful. AFB isolated from 77 (29.8%) samples. One hundred and sixty (62%) samples were smear and culture negative and the remaining samples 21 (8.1%), were contaminated (Table 1). According to phenotypic tests, from 77 NTM species, 52 (70.1%) strains of rapid growing mycobacteria (RGM), and 25 (32.4%) strains of slowly growing mycobacteria (SGM) were detected. Table 1 represents the detailed distribution of water samples from hospitals in three cities of Khuzestan province. *M. fortuitum* complex-like group was the most frequently encountered species (*n* = 40, 51.9%), followed by *M. gordonae*-like organisms (*n* = 13, 16.8%) and *Mycobacterium avium* complex (*n* = 5, 6.4%). The remaining isolates were unidentifiable with phenotypic evaluation (*n* = 16, 20.7%). By using *hsp65*-PRA, *M. fortuitum* was the most frequently encountered species (*n* = 34, 44.1%), followed by *M. gordonae*, (*n* = 13, 16.8%). The remaining 30 isolates, were mostly showed identical PRA pattern with other species or unique and unknown patterns in comparison to patterns deposited in free available database (http://app.chuv.ch/prasite). For more precise identification and reliable access to species spectrum of the NTM isolates, randomly selected isolates from each cluster of *hsp65*-PRA, were subjected to sequence based identification using *rpoB* gene. NTM isolates from all clusters except two (SD31 and SD51), were confidently identified by *rpoB* gene. The latter two strains were clearly differed from any other known *Mycobacterium* species and displayed the best resemblance with *M. arupense* and *M. wolinsky,* respectively. The results of NTM identification by phenotypic tests, PRA and sequence analysis are presented in details in Table 2. Several clusters were characterized in RGM and SGM groups by phylogenetic constructed tree using *rpoB* gene. The isolates SD31 and SD51 characterized with separated clusters with high bootstrap percentages, representing distinct species (Figure 1). The frequency of NTM species in different hospital water sources and geographic distribution of these species among the hospitals of three cities of Khuzestan, are presented in Table 3; Figure 2, respectively.

**Table 1:** **Distribution of water samples from hospitals in different cities of Khuzestan, Iran**.

**Location**	**Water samples (*****n*** = **105)**			**Total**
	**Positive**	**Negative**	**Contaminated**	
Ahvaz	47 (26.1%)	115 (63.8%)	18 (10%)	180 (40%)
Abadan	17 (42.5%)	22 (55%)	1 (2.5%)	40 (12.3%)
Dezful	13 (34.2%)	23 (60.5%)	2 (5.2%)	38 (13.3%)

**Table 2:** **Results of NTM identification by phenotypic and molecular tests using combination of phenotypic and molecular methods**.

**Number of isolates**	**Identification by**		
	**Phenotypic tests**	**PRA**	***rpoB* gene sequence**
34	*M. fortuitum complex*	*M. fortuitum*	*M. fortuitum*
13	*M. gordonae like*	*M. gordonae group*	*M. gordonae*
4	*Mycobacterium sp*	*M. phocaicum or M. mucogenicum*	*M. phocaicum*
6	*M. fortuitum complex*	*M. conceptionense or M. senegalense*	*M. senegalense*
3	*M. simiae like*	*M. lentiflavum/M. simiae*	*M. simiae*
5	*M. avium complex*	*M. colombiense/M. avium subsp. avium*	*M. colombiense*
4	*Mycobacterium sp*	*M. lentiflavum*	*M. lentiflavum*
3	*Mycobacterium sp*	*M. novocastrense*	*M. novocastrense*
2	*Mycobacterium sp*	unknown	*M. engbaekii*
1	*Mycobacterium sp*	unknown	*M. iranicum*
1	*Mycobacterium sp*	*M. arupense*	Unidentified
1	*Mycobacterium sp*	unknown	Unidentified

*PRA, PCR restriction enzyme analysis*.

**Table 3:** **Frequency of NTM isolates from different hospital water sources**.

**Water sources**	**Number (%) of samples**	**Number (%) of positive samples**	**NTM species (based on analysis)**
Hospital Tap water	135	26	*M. fortuitum* (12), *M. gordonae* (6), *M. phocaicum* (3), *M. senegalense* (2), *M. engbaekii* (2), Unidentified (1)
Dentistry units water	54	22	*M. fortuitum* (13), *M. gordonae* (2), *M. iranicum* (1), *M. colombiense* (3), *M. lentiflavum* (1), *M. novocasterense* (1), *M. phocaicum* (1)
Haemodialysis center	17	8	*M. fortuitum* (3), *M. novocastrense* (2), *M. senegalense* (2), *M. lentiflavum* (1)
Ventilator water	25	7	*M. simiae* (3), *M. colombiense* (2), *M. fortuitum* (2)
Water tank	27	14	*M. fortuitum* (4), *M. gordonae* (5), *M. senegalense* (2), *M. lentiflavum* (2), Unidentified (1)

## Discussion

In present study, we applied a combination of phenotypic and molecular methods for the detection and identification of NTM species in hospital water samples. Totally, AFB were isolated from 77 (29.8%) samples, of which, 52 (70.1%) were RGM, and 25 (32.4%) strains were SGM. Our recovery percentage is lower than other investigations on hospital waters (Shin et al., 2007, 2008; Hussein et al., 2009; Fernandez-Rendon et al., 2012). This may be explained by the lower number of examined samples by them compared to our study. Our findings confirmed that species identification by *hsp65*-PRA was significantly more accurate than the phenotypic methods, as other investigators reported (Martin et al., 2007; Chimara et al., 2008). The limitations associated with phenotypic tests and PRA, such as the presence of unknown patterns, and species with identical patterns, highlights the need for more reliable identification methods (Moghim et al., 2012). Sequence based methods offer much more resolution in identification of NTM compared to other assays (Adékambi et al., 2003). Based on the *rpoB* gene sequences analysis, 75 (97.4%) isolates were identified correctly to the species level.

In current study, *M. fortuitum* and *M. gordonae* were the most common inhabitant species recovered from hospital water samples. Recovery of these two species clearly demonstrated that these are the core of culturable microbial community living at different water distribution systems. However, the occurrence of each NTM species at different points of hospital water distribution systems, remained to be investigated in future studies. In one hand, two species of *M. fortuitum* and *M. gordonae* were the most frequently recovered species from all three parts of Khuzestan province (Figure 2), indicating that there was a distinct and identical distribution pattern among the hospital water samples. on the other hand, two reports from this part of Iran, showed that *M. fortuitum* was the dominant potential pathogen recovered from clinical samples (Khosravi et al., 2009; Hashemi-Shahraki et al., 2013), highlights the role of water as bridge between environment and disease caused by NTM (Mirsaeidi et al., 2014). Other reports from Iran, have demonstrated *M. fortuitum* as the dominant species both in water and clinical samples (Shojaei et al., 2011; Moghim et al., 2012; Nasr-Esfahani et al., 2012; Hashemi-Shahraki et al., 2013). In contrast, in several different studies, *M. gordonae, M. kansasii*, and *M. chelonae* were shown to be the most prevalent mycobacterial species in water supplies of hospitals (Sebakova et al., 2008; Shin et al., 2008; Hussein et al., 2009; Montanari et al., 2009).

In this study, several species of potential pathogenic NTM such as *Mycobacterium simiae* were also recovered. *M. simiae* pseudo-outbreaks had been reported by El Sahly et al. (2002), from a community teaching hospital in Houston, Texas. In their report, contaminated hospital water supply was identified as a source of human outbreak. Hashemi-Shahraki et al. (2013), have been reported *M. simiae* as emerging pathogen from Iran, with no person to person transmission. In such cases, water might be served as main source of human infection.

In current study, from 54 dental units, we recovered 22 NTM species including *M. fortuitum*, Mycobacterium *colombiense*, and *M. gordonae*. Schulze-Röbbecke et al. (1995), were reported the isolation of *M. gordonae, M. flavescens, M. chelonae, M. chelonae*-like organism, and *M. simiae* from dental units. This finding leaded to conclusion that, high numbers of NTM may be swallowed, inhaled or inoculated into oral wounds during dental treatment and in some case resulting in colonization, sensitization or infection.

Dental unit waterline usually contaminated with the respiratory pathogens such as *Legionella* spp., *Mycobacterium* spp., and *Pseudomonads*. Nevertheless, in order to decrease infection risk, less than 200 colony-forming units (CFU) per ml of aerobic bacteria should be considered as a standard of the dental unit water (Pankhurst, 2003). Although, CFU of mycobacteria in dental units was not evaluated in our study, but recovery of potential pathogenic NTM from the dental water samples confirmed the high risk of NTM infection in individuals undergo dental procedures. Despite high aerosolization of NTM during dental treatment, a minimum rate of infection and sensitization risks associated with NTM, was reported (Dutil et al., 2007). Similar to our study, frequent isolation of NTM from dental units in different parts of the world.

(Schulze-Röbbecke et al., 1995; Pankhurst, 2003; Walker et al., 2004; Dutil et al., 2007), emphasizes the need for effective mechanisms to reduce NTM burden within dental unit systems, and highlights the risk of occupational exposure and infection in general dental practice.

A fairly diverse potential pathogenic NTM species was recovered in our study including *M. colombiense*, which is belong to the *M. avium* complex (MAC). This species is responsible for lymphadenopathy in immunocompetent children in Spain (Esparcia et al., 2008), and France (Vuorenmaa et al., 2009). In this study, we recovered this species form dental units and ventilator water samples. This is the first report for isolation of this species from Iran.

Falkinham (2010), reported high numbers of *M. avium* isolates from bronchoscopes and the filters used for washing them in USA. In their study, from water and biofilm samples collected from the bronchoscopies, *M. avium, M. intracellulare, M. malmoense*, and *M. gordonae* recovered, showing the contamination of bronchoscopes with water supply.

Our results showed high diverse patterns for 35% (27 isolates) saprophytic mycobacteria and 50% (50 isolates) potentially pathogenic mycobacteria. In compare, in a report from Korea, half of the tap water samples (50 of 100) from different parts of the hospital were positive for mycobacteria including saprophytic mycobacteria (73.3%), potentially pathogenic mycobacteria (21.7%) and unidentifiable strains 5% (Shin et al., 2007).

In Turkey, Genc et al. (2013) examined 160 water samples from various departments of two hospitals in Istanbul. Based on their report, NTM were detected in 33 (20.6%) samples, and *M. lentiflavum, M. gordonae*, and *M. peregrinum*, were the most common NTM isolated in rates of 60.6, 30.3, and 9.1%, respectively. In concordant to their report, we demonstrated a rate of 29.8% NTM from water samples, however the NTM species diversity including RGM, was higher in our study compared to them.

Fernandez-Rendon et al. (2012), in Mexico City, reported potable water samples including cistern, kitchen tap, and bathroom showers harbored NTM species mostly *Mycobacterium mucogenicum*, while *M. rhodesiae, M. peregrinum,* and *M. fortuitum* were recovered as rare species. Several reports from other parts of the world (Walker et al., 2004; Hussein et al., 2009; Briancesco et al., 2014; Crago et al., 2014) showed that contamination of water resources in hospital, is not restricted to poor setting. Although, our setting as a poor one, showed high contamination rate and more diverse potential pathogenic species of NTM.

Contamination of hemodialysis fluid with whole cell of bacteria or their fragments is a significant health risk for hemodialysis patients (Glorieux et al., 2009). In our study, from 17 hemodialysis water samples, we recovered 8 NTM species including *M. fortuitum* (*n* = 3), *M. novocastrense* (*n* = 2), *M. senegalense* (*n* = 2), and *M. lentiflavum* (*n* = 1). In line with our findings, high levels of contamination in dialysis water fluid were reported in different medical settings. At hemodialysis centers in nine hospitals in Japan, Oie et al. (2003), reported high contamination of dialysate. Of 40 dialysate samples analyzed, 42.5% showed a bacterial count of more than 2000 CFU/ml, which was above the standard. In another similar study conducted in the Brazil, 11 mycobacterial species (*M. gastri, M. kansasii, M. lentiflavum,* and *M. gordonae*) were identified from 19 hemodialysis machines (Montanari et al., 2009). Moreover, in a recent study from Iran, from 80 samples originated from hemodialysis distribution system of the hospitals under investigation, a diverse bacterial community, including Mycobacteria, was detected (Heidarieh et al., 2016).

Due to the risk of NTM-contaminated hospital water supplies for nosocomial infections, preventive measures may be considered in hospitalized patients particularly whom suffering from immunosuppression conditions. Hospital potable water harboring saprophytic and potentially pathogenic NTM represents a potential source for nosocomial infections.

In conclusion, the findings of the present study suggest that, water is an important environmental source harboring NTM. Furthermore, the wide presence of NTM in the aquatic sources throughout Khuzestan, Iran, is a potential public health hazard especially for those with immunodeficiency, calls for more effective water disinfection procedures in these units. A wide range of sources, exposures, and modes of transmission need to be investigated and additional studies are needed to correlate patient and environmental isolates. This is where the genetic identification using molecular techniques becomes useful since they can increase the specificity, sensitivity and the accuracy of the diagnosis.

## Author contributions

AK: Substantial contributions to the conception or design of the work; Final approval of the version to be published; Agreement to be accountable for all aspects of the work in ensuring that questions related to the accuracy or integrity of any part of the work are appropriately investigated and resolved. AS: Substantial contributions to the conception or design of the work; Final approval of the version to be published; Agreement to be accountable for all aspects of the work in ensuring that questions related to the accuracy or integrity of any part of the work are appropriately investigated and resolved. MH: Substantial contributions to the conception or design of the work; Drafting the work or revising it critically for important intellectual content; the acquisition, analysis, and interpretation of data for the work. RM: acquisition, analysis, interpretation of data for the work; Final approval of the version to be published. AT: acquisition, analysis, interpretation of data for the work; Agreement to be accountable for all aspects of the work in ensuring that questions related to the accuracy or integrity of any part of the work are appropriately investigated and resolved.

### Conflict of interest statement

The authors declare that the research was conducted in the absence of any commercial or financial relationships that could be construed as a potential conflict of interest.
